# Universal cancer screening: revolutionary, rational, and realizable

**DOI:** 10.1038/s41698-018-0066-x

**Published:** 2018-10-29

**Authors:** David A. Ahlquist

**Affiliations:** Mayo Clinic, Rochester, MN USA

## Abstract

Cancer remains the second leading cause of mortality worldwide, and overall cancer-related deaths are increasing. Despite the survival benefit from early detection, screening has to date targeted only those few organs that harbor tumors of sufficient prevalence to show cost-effectiveness at population levels, leaving most cancer types unscreened. In this perspective overview, a case is made for universal cancer screening as a logical and more inclusive approach with potentially high impact. The centrally important conceptual drivers to universal screening are biological and epidemiological. The shared biology of tumor marker release into a common distant medium, like blood, can be exploited for multi-cancer detection from a single test. And, by aggregating prevalence rates, universal screening allows all cancers (including less common ones) to be included as targets, increases screening efficiency and integration across tumor types, and potentially improves cost-effectiveness over single-organ approaches. The identification of new tumor marker classes with both broad expression across tumor types and site-prediction, remarkable advances in assay technologies, and compelling early clinical data increase the likelihood of actualizing this new paradigm. Multi-organ screening could be achieved by targeting markers within or stemming from the circulation (including blood, urine, saliva, and expired breath) or those exfoliated into common excretory pathways (including the gastrointestinal and female reproductive tracts). Rigorous clinical studies in intended use populations and collaborations between academia, industry, professional societies, and government will be required to bring this lofty vision to a population application.

## Introduction

Cancer exacts an alarming toll. Cancer remains the number one cause of death in the U.S. among those younger than 80;^1^ it is the second leading cause of mortality worldwide accounting for roughly 1 of every 6 deaths.^2^ Despite encouraging drops in mortality rates with some cancers due to earlier detection and improved treatment,^1^ overall cancer deaths are increasing globally.^1,2^ Importantly, pre-symptomatic screening is associated with earlier stage diagnosis and improved outcomes.^3,4^ However, most cancer types are not currently targeted for whole population screening^5^ and, consequently, present symptomatically and typically at late and more difficult to cure stages;^1,6^ as examples, unscreened cancers of the lung, pancreas, esophagus, stomach, and ovary have regional or distant metastases in the majority of cases at the time of diagnosis.^1,6^ Filling this void in cancer control could have a potentially enormous impact on morbidity and mortality reduction. It is only through effective pre-symptomatic population-wide screening that a meaningful shift toward early-stage cancer detection can be achieved.

This brief overview perspective makes the case for universal cancer screening as a logical advance beyond the current single-organ approach. A universal screening strategy is supported by strong biological and epidemiological rationale, and its achievability is increasingly likely based on emerging high performance technologies with compelling early data. Both academia and industry are now actively pursuing approaches to achieve the lofty goal of universal cancer screening.

## The single-organ screening approach: inherent limitations

Cancer screening has evolved historically using tools that target single organs. Current guidelines by the American Cancer Society recommend population-wide screening in those at average risk for just four cancers—breast, cervix, colorectum, and prostate.^5^ However, such general population screening has not been justified or recommended for most cancer types due primarily to individual prevalence rates that are insufficient to allow cost-effective interventions using a single organ approach.

Single-organ strategies to increase prevalence by targeting only the high-risk subsets have been pursued with several cancer types. For example, pancreatic cancer screening may be recommended in those with a strong family history,^7^ lung cancer screening is endorsed for those with a history of heavy smoking,^8^ and hepatoma screening is applied to those with known chronic liver disease.^9^ Yet, while these three cancer types have among the highest mortality rates in the U.S. and other countries,^1,2^ none is screened at the population-wide level where many or most cancer deaths from each occur.

**Fig. 1:**
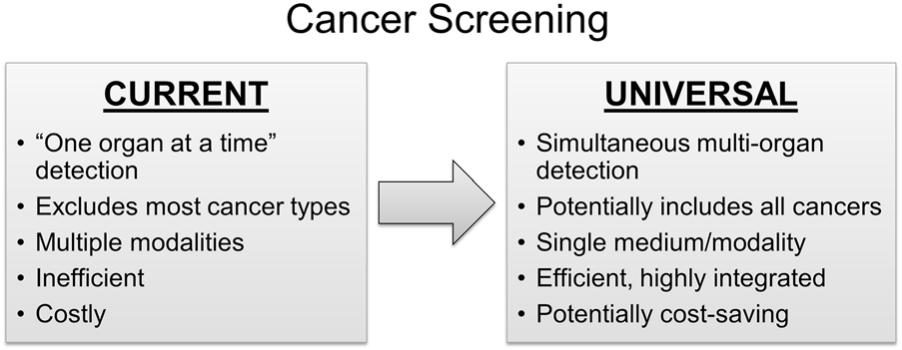
Current single-organ and future universal cancer screening approaches: a conceptual comparison of features

In addition to the exclusion of lower-prevalence cancers with this traditional approach, single-organ screening has relied on disparate screening modalities and preparations which may challenge integration, reduce scheduling efficiency, compromise compliance, and increase logistical costs overall (Fig. 1).

## Universal cancer screening: re-imagining the paradigm

Universal cancer screening is a conceptually intriguing approach to fill the current gap. In fact, a multi-organ approach may be the only logical strategy to screen lower prevalence cancers in a cost-effective manner, and it does so by simultaneously targeting multiple tumor types and aggregating their prevalence rates.

There are several important conceptual advantages that a universal cancer screening approach brings (Fig. 1). In contrast to single-organ screening, the “universe” of cancers is screened and the totality of organ systems rather than an individual organ becomes the screening target and denominator for performance metrics. As a universal screening tool might be performed non-invasively on a single medium (e.g., blood, urine, saliva, breath, or other) without the need for lengthy preparations or time away from work, this approach could dramatically improve screening efficiency, integration across tumor types, and patient compliance. While robust cost-effectiveness models will need to be created and analyzed, a universal approach has potential to be cost-saving from vantage points of patients, society, and third-party payers.

### The power of aggregate prevalence

The efficiency, cost-effectiveness, and potential impact of screening are all directly related to cancer prevalence. Cancer prevalence could be defined as the proportion of persons within a population who have cancer at a point in time, and it is a measure that combines tumor incidence and pre-diagnostic dwell time. Prevalence estimates vary widely based on methods used and on age and other demographic factors in populations studied.^10^ Extrapolations from autopsy series suggest that roughly 7–11% of those aged 50–75 harbor an internal malignancy,^11–13^ and that cancer may be the unsuspected cause of death in 3–5%.^11,12^

**Fig. 2:**
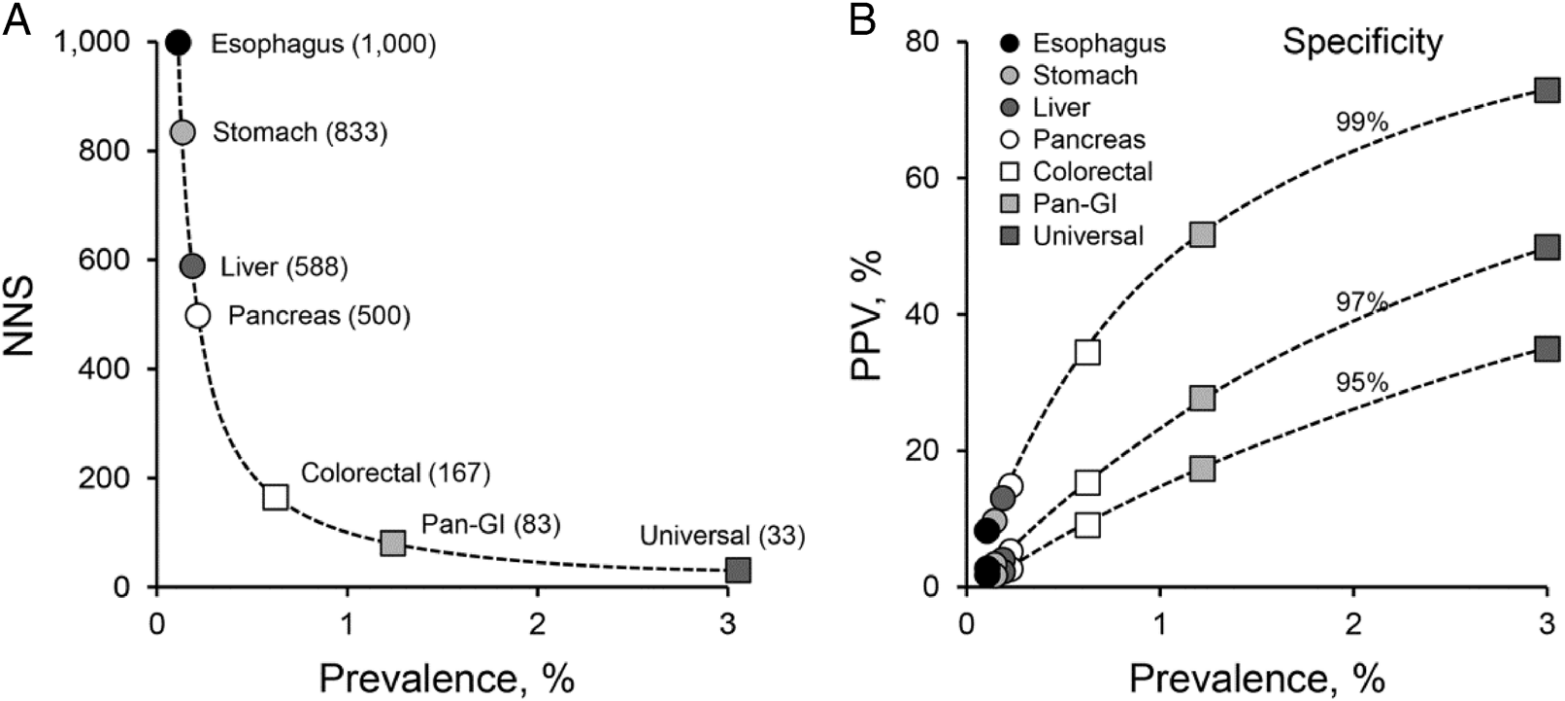
Impact of cancer prevalence on screening efficiencies. **a** Exponential relationship between cancer prevalence and the number of patients needed to be screened to detect a single cancer (NNS). Estimated NNS is plotted for cancers at individual gastrointestinal organs (only colorectal screening is currently practiced), for combined gastrointestinal cancers (Pan-GI), and for all cancer types in aggregate (Universal). For this illustration, detection sensitivities of 100% were assumed in calculations of NNS. **b** Influence of cancer prevalence on positive predictive value (PPV) at various specificities. For illustrative purposes, estimated PPVs are plotted for same spectrum of single and combined cancer screening approaches as in **a**. For both **a** and **b**, conservative prevalence estimates obtained from the literature are used.^1,11,12^

To illustrate the striking influence that aggregated tumor prevalence has on measures of screening efficiency, we can consider gastrointestinal (GI) cancers as a multi-organ cluster (Fig. 2). Among the major GI cancers, only colorectal cancer (CRC) is sufficiently prevalent to justify population-wide screening from a single-organ approach. The estimated number of persons needed to be screened to detect one cancer (NNS) is about 167 for CRC,^14^ but the estimated NNS increases exponentially for the other less prevalent GI cancers ranging from approximately 500 with pancreatic cancer to 1000 with esophageal cancer even with perfect test sensitivity (Fig. 2a). However, if all major GI cancers are targeted in aggregate, the estimated NNS falls to just 83. And, this compares to an estimated NNS of only 33 (using a conservative overall cancer prevalence estimate of 3%) if the universe of cancers is targeted. Furthermore, the probability that a positive screening test indicates the presence of a tumor (positive predictive value (PPV)) not only increases with test specificity but is also markedly affected by tumor prevalence (Fig. 2b). Aggregating prevalence rates of GI cancers in a pan-GI test or of all cancers in a universal test yield much higher PPVs than by single-organ screening approaches to individual GI cancers. The combination of low NNS and high PPV translates to high impact value of a screening intervention.

## Non-invasive approaches to multi-organ cancer screening

An ideal tool for universal or multi-organ screening would be highly sensitive for detection of curable stage disease across tumor types to achieve optimal effectiveness, be highly specific to limit false-positives and enhance PPV, and accurately predict tumor site to efficiently direct the diagnostic evaluation of those with a positive test result. In addition to these ideal performance characteristics, desirable features would include non-invasiveness, ready distribution, and affordability to encourage patient compliance and unfettered access. While there are no established methodologies that currently meet these criteria, promising new candidate tools with potential for multi-organ cancer detection are emerging.

### Imaging with clearer vision

Whole body imaging, such as by CT scanning, has been considered historically as an approach to universal cancer screening. However, formal prospective studies have not been pursued due, in part, to concerns that early iterations of such tools lacked sufficient sensitivity or specificity for multi-organ cancer screening and could lead to potentially harmful side effects.^15,16^

Innovative and more accurate next-generation approaches could re-open doors for universal screening by imaging in the future. Various molecular, nano-particle, and fluorescent constructs have been combined with ultrasound, magnetic resonance, optical, photoacoustic, and other imaging modalities to yield novel cancer detection approaches with potential for extremely high resolution.^17–19^ While these new approaches may have initial use in diagnosis and surveillance, their absence of ionizing radiation and capacity for whole body imaging makes them intriguing candidates for potential multi-organ screening. Further technical refinements and rigorously conducted clinical studies in appropriate target populations are needed to assess their safety, accuracy, and broad feasibility in a universal cancer screening application.

### A cornucopia of markers and analytical techniques

A diverse array of tumor marker types now provides excellent candidates for multi-organ screening, some with promisingly high discrimination and site-specificity. Candidate marker categories range from whole tumor cells, to characteristic constituents of tumor cells (such as genetically or epigenetically altered DNA, qualitative and quantitative changes in RNA species, and various proteins), to host response elements (e.g., auto-antibodies), and even to metabolite profiles. For site prediction, epigenetic markers (e.g., aberrantly methylated DNA and nucleosomal changes) are particularly attractive choices based on their biological role in tissue differentiation and early data in plasma and stool showing patterns highly associated with tumor location.^20–23^

Novel assay tools are at or approaching the requisite analytical sensitivity for marker detection in distant media, even at the low abundance levels typically seen with early stage neoplasia.^24–29^ Additionally, sophisticated analytical software techniques, such as machine learning and various forms of artificial intelligence,^30,31^ are capable of recognizing discriminant diagnostic patterns within complex data sets that would otherwise be difficult to identify and are increasingly being applied to molecular diagnostics.

Because of the molecular and phenotypic heterogeneity of tumors within and across sites, the most informative marker panels may well need to encompass multiple marker classes to optimize detection accuracy, as has been done combining DNA and protein markers in plasma^24^ and in stool.^14,32^

### Targeting the circulation: a marker depot common to all tumors

The circulation represents a dump site shared by essentially all internal malignancies. While tumor cells may gain access to the circulation by direct vascular invasion, a variety of other potential biological mechanisms may permit passage of tumor elements into the circulation prior to invasion, including necrosis or apoptosis, micro-vesicle budding,^33^ and phagocytosis,^34^ and contribute candidate targets for effective detection of earliest stage disease. Host responses, such as by formation and release of auto-antibodies, may also be reflected in the circulation and serve as potential early stage detection markers.^35^ Exploiting this biology common to all internal cancers, the circulation provides a most logical and strategic target for universal cancer screening.

#### Blood

**Fig. 3:**
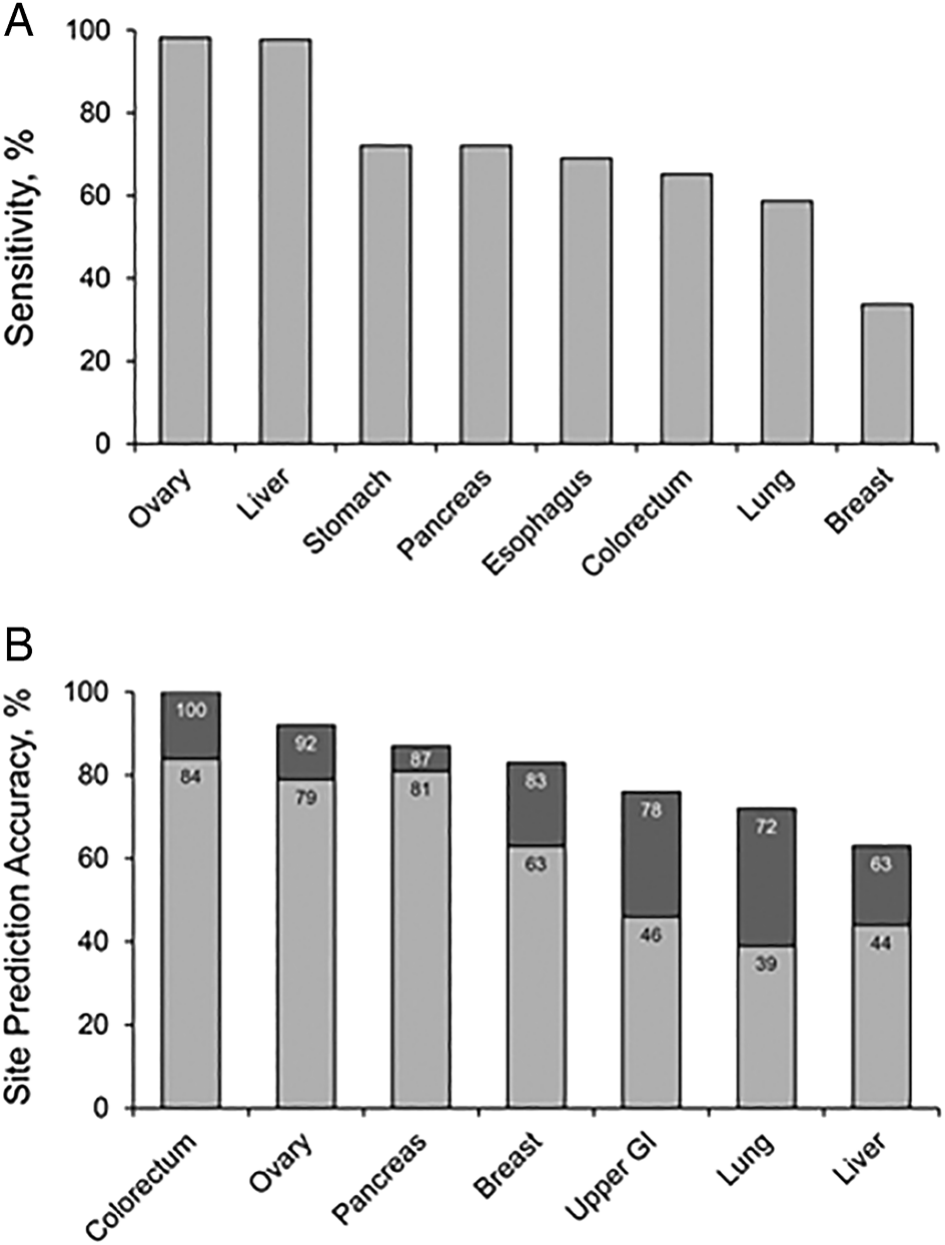
Detection and site prediction of surgically resectable cancers with a multi-analyte blood test: early results. Performance data from a prototype assay targeting various proteins and gene mutations in plasma are shown across eight common cancer types. **a** Sensitivity by cancer type at 99% specificity. **b** Accuracy of tumor localization in test-positive patients. Percentages correspond to the proportion of patients in whom tumor location was correctly classified as the most likely site (light bars) or as one of the two most likely sites (light + dark bars). Figures modified from the original publication.^24^

Blood is the medium directly within the circulation, and the plasma and serum have been most studied. Plasma cell-free nucleic acids, both DNA and RNA, have been studied extensively as cancer detection markers.^36^ While assays of nucleic acids alone have generally yielded lower detection rates with earliest stages of most cancer types, high detection rates across all stages have been achieved with some tumors, as in recent studies on hepatocellular cancer.^37^ Several groups have demonstrated that genome sequencing platforms applied to plasma are capable of multi-organ cancer detection. In a recent report based on the combined assay of gene mutations and a panel of historical cancer-associated proteins in plasma,^24^ investigators demonstrated that multiple cancer types could be detected (Fig. 3a); furthermore, tumor site could be predicted in test-positive patients with moderate to high accuracy (Fig. 3b).

Various compartments within blood may contain tumor-derived materials that could serve as potentially valuable screening targets. For example, circulating micro-vesicles may house discriminant tumor-specific RNA, proteins, and other compounds and prevent their enzymatic breakdown within the circulation.^33^ Circulating large macrophages containing tumor cells or cell debris have been described with multiple cancer types and across tumor stages.^34,38^ And, a novel blood-based detection approach involving exogenously delivered, genetically encoded mini-circle reporters is being developed to produce tumor-driven biomarkers with potential high sensitivity and specificity for multiple cancers.^39^

Major initiatives are underway commercially using diverse approaches, and large clinical studies targeting intended use populations with refined assay techniques will be forthcoming.

#### Indirect media

Other media (e.g., urine, saliva, and breath) contain materials arising from the circulation and may also be interrogated non-invasively as potential sources of tumor markers for multi-organ screening. While much research is needed for corroboration, recent early phase data using novel markers and technical approaches on each medium show promise. Marker degradation and limited fragment size allowed by the glomerular filter have been historical impediments to use of urine as a targeted medium for pan-cancer screening. However, a new nano-wire device embedded in a micro-fluidic system appears to be an efficient method to capture extracellular vesicles that contain preserved tumor-specific RNA signatures which can potentially be applied to multi-organ screening.^40^ Exciting innovations have emerged to assay circulatory markers in saliva, including nucleic acids, proteins, and metabolites, with potential to be used in multi-organ screening.^41^ And, sophisticated “electronic nose” devices have been developed to analyze volatile organic compounds in exhaled breath; this approach has revealed characteristic metabolic patterns of potential value in detecting multiple cancers including lung, liver, colorectal, breast, ovarian, gastric, and head and neck.^42,43^

### Capitalizing on tumor exfoliation

There are several anatomic corridors of organs connected in series or by appendage that exfoliate surface cells into a common route of efflux yielding a single excretory medium that can be interrogated for the aggregate detection of multiple tumor types. Best examples of anatomic corridor systems are the GI tract and female reproductive tract. As luminal exfoliation occurs from both precursor lesions and earliest stage cancers before vascular invasion has occurred, it follows that this mechanism of marker release may allow for detection of these important neoplastic targets prior to vascular invasion.^44^

#### By stool

It is now well-established that CRC and advanced precursor lesions can be detected with high accuracy using a multi-target stool DNA test, achieving detection rates for early stage cancer essentially equivalent to those of colonoscopy.^14,32,45^ Early data suggest that the value of stool testing can be expanded to include detection and site prediction of the historically unscreened upper GI cancers as well.^23^ A challenge with this approach is the harsh digestive gauntlet that exfoliated markers must traverse and survive; and targeting partially digested short fragment analytes may be one way to mitigate this effect.^46^ Assay optimization and rigorous clinical testing are clearly needed to adequately assess this pan-GI screening approach.

#### By tampon

Given the normal physiology of the female reproductive tract with cyclic passage of ova from the ovaries down the fallopian tubes into the uterus and the regular shedding of uterine endometria into the vagina, it follows that any gynecological neoplasm (ovarian, endometrial, and cervix) may exfoliate cells or cell debris that could be recovered from a vaginal pool sample as a potential approach to multi-organ screening. Indeed, proof-of-concept for detection of endometrial cancer via assay of methylated DNA markers extracted from a vaginal tampon has been established.^47^ Furthermore, early data show that both endometrial and ovarian cancers can be detected by molecular analysis of cervical fluids collected during a routine cervical Papanicolaou test.^48^ Thus, the biology and early clinical observations support the concept of a tampon device to simultaneously screen gynecological neoplasms in aggregate. Further technical refinements and clinical studies are needed to establish the value of this approach.

## Future challenges and uncertainties

While early data are most encouraging and the potential value of universal cancer screening is high, there’s much work and several categorical challenges ahead. First, the performance of optimized tests will need to be validated in well-designed clinical studies that target intended-use populations. Second, the potential for undesirable screening outcomes, such as “over-diagnosis” (detection of indolent cancers that would not harm persons during their lifetimes) and “false-positives” (test positive results in absence of cancer) must be considered at the front end of this effort. Tests should be intentionally engineered to mitigate or minimize such outcomes through marker selection, setting of high specificity cutoffs, and other means. As above (Fig. 2b), the combination of high specificity and high aggregate tumor prevalence potentially leads to PPVs that far exceed those seen with current single-organ screening. Third, these new tests, for which there is no predicate, will need to reviewed and approved by regulatory agencies, third party payers, and groups that recommend clinical practice guidelines. Along with solid technical and clinical data, robust cost-effectiveness modeling and well-considered clinical algorithms should facilitate these processes. And, finally, it remains to be determined if a universal or multi-organ cancer screening test would be of greatest value as a complement to or replacement of current single-organ approaches. The strength of emerging data at individual organ levels will help to make this judgment and engineer best systems.

## Summary

Multi-organ cancer screening could be transformational and fill an enormous existing gap in cancer control. Taking advantage of aggregate tumor prevalence with shared marker deposition into a common distant medium and of assays with the capacity to predict tumor site, a single multi-marker test could potentially provide both “universalized” value by detecting all cancer types and “individualized” value by tailoring the evaluation of a positive test to the likely organ of origin. Increasingly accurate tumor markers and marker panels along with an array of high performance new assay tools show great promise. Substantial collaborative efforts between academia, industry, professional societies, and government will be needed to successfully bring this revolutionary cancer screening approach to the population.
